# Correction: A natural history study to track brain and spinal cord changes in individuals with Friedreich’s ataxia: TRACK-FA study protocol

**DOI:** 10.1371/journal.pone.0320683

**Published:** 2025-03-18

**Authors:** Nellie Georgiou-Karistianis, Louise A. Corben, Kathrin Reetz, Isaac M. Adanyeguh, Manuela Corti, Dinesh K. Deelchand, Martin B. Delatycki, Imis Dogan, Rebecca Evans, Jennifer Farmer, Marcondes C. França, William Gaetz, Ian H. Harding, Karen S. Harris, Steven Hersch, Richard Joules, James J. Joers, Michelle L. Krishnan, Michelle Lax, Eric F. Lock, David Lynch, Thomas Mareci, Sahan Muthuhetti Gamage, Massimo Pandolfo, Marina Papoutsi, Thiago J. R. Rezende, Timothy P. L. Roberts, Jens T. Rosenberg, Sandro Romanzetti, Jörg B. Schulz, Traci Schilling, Adam J. Schwarz, Sub Subramony, Bert Yao, Stephen Zicha, Christophe Lenglet, Pierre-Gilles Henry

There are errors in the Funding statement. The correct Funding statement is as follows: This study is funded by grants from the Friedreich’s Ataxia Research Alliance (FARA) to each of the academic sites and IXICO plc with financial support from Takeda Pharmaceuticals Company Ltd, Novartis Gene Therapies, IXICO plc and PTC Therapeutics. The Friedreich Ataxia Research Alliance does not use grant numbers. Monash University is the coordinating site. The study sites and the site principal Investigators (main authors who received funding) are detailed below. Monash University (N.G.K) University of Minnesota (P.G.H and C.L) Children’s Hospital of Philadelphia (W.G) University of Florida (S.S) RWTH Aachen University (K.R) University of Campinas (M.C.F) McGill University (M.Pan) Funding bodies Friedreich’s Ataxia Research Alliance (FARA): https://www.curefa.org/IXICOplc:https://ixico.com/ Novartis Gene Therapies: https://www.novartis.com/about/innovative-medicines/novartis-pharmaceuticals Takeda Pharmaceuticals company Ltd: https://www.takeda.com/en-au/ PTC Therapeutics:https://www.ptcbio.com/ FARA plays an ongoing role in study oversight, study design, decision to publish and author J.F who is J.F. is employed by the Friedreich’s Ataxia Research Alliance (FARA) played a role in the preparation of the manuscript. Takeda Pharmaceuticals Company Ltd plays an ongoing role in study oversight, study design, decision to publish and author S.Z is an employee of Takeda Pharmaceuticals Company Ltd and played a role in preparation of the manuscript. Additionally former employees of Takeda Pharmaceuticals. A.J.S. and R.E. also contributed to the preparation of the manuscript. PTC Therapeutics plays an ongoing role in study oversight, study design, decision to publish and authors T.S. and B.Y. who are employees of PTC Therapeutics contributed to the preparation of the manuscript. Novartis Gene Therapies plays an ongoing role in study oversight, study design, decision to publish and authors M.L.K who holds shares in Novartis Gene Therapies as indicated in the conflicts of interest played a role in the preparation of the manuscript. IXICO plc plays an ongoing role in study oversight, study design, decision to publish will perform independent quality control and data analysis of brain anatomical imaging and brain diffusion imaging data for one third of TRACK-FA participants. Authors M.L, R.J and M.Pap are employees of IXICO and contributed to the preparation of the manuscript.

There are errors in the Competing Interests statement. The correct Competing Interests statement is as follows: I have read the journal’s policy and the authors of this manuscript have the following competing interests: S.Z. is employed by Takeda Pharmaceutical Company Ltd and receives salary and holds stocks in the company. A.J.S. and R.E were employed by Takeda Pharmaceutical Company Ltd at the time of their contribution to the TRACK-FA project. Takeda Pharmaceutical Company Ltd remains committed to FRDA research and will help develop translational tools to monitor patient disease and share with the FRDA community. T.S. and B.Y. are both employees of PTC Therapeutics. D.L. is a grant recipient from the National Institute of Health (NIH), Muscular Dystrophy Association (MDA), Friedreich’s Ataxia Research Alliance (FARA), Reata Pharmaceuticals Inc, Retrotope Inc, Voyager Therapeutics, Novartis Gene Therapies, Audentes Therapeutics (Astellas Gene Therapies) and Minoryx Therapeutics S.L. T.P.L.R. has equity interest in PRISM Clinical Imaging and Proteus Neurodynamics and consulting/advisory board engagement with CTF MEG International Services LP, Ricoh Company Ltd, Spago Nanomedical AB, Avexis (Novartis Gene Therapies) and Acadia Pharmaceuticals Inc. P.G.H. is a grant recipient from the Friedreich’s Ataxia Research Alliance (FARA), GoFAR, Ataxia UK, the Bob Allison Ataxia Research Centre, and the National Institute of Health (NIH). CMRR is supported by NIH grants P41EB027061 and P30NS076408. P.G.H. reports grants from Minoryx Therapeutics for activities outside this study. M.Pap. and R.J. and M.L. are employed by IXICO plc, ML is a shareholder for IXICO plc. M.C.F. is a grant recipient from PTC Therapeutics and has taken part in advisory board for PTC Therapeutics and Avexis (Novartis Gene Therapies). T.J.R.R. is a grant recipient from the Friedreich’s Ataxia Research Alliance (FARA). C.L. is a research grant recipient from the Friedreich’s Ataxia Research Alliance (FARA), GoFAR, Ataxia UK, the Bob Allison Ataxia Research Center, and National Institute of Health (NIH) grants P41. EB027061 and P30 NS076408. C.L. reports research grants from Minoryx Therapeutics and Biogen Inc. for activities outside this study. S.S. is a broad member of the Research Advisory Board for National Ataxia Foundation (USA), a research grant recipient from the Friedreich’s Ataxia Research Alliance (FARA), Wyck Foundation, National Ataxia Foundation, Muscular Dystrophy Association (MDA), National Institute of Health (NIH), FDA and receives industry support from Reata Pharmaceutical Inc, Retrotope Inc, PTC Therapeutics, Biohaven Pharmaceuticals, Avidity Biosciences Inc, and Strides Pharma Science Limited. M.L.K. holds shares in Novartis Gene Therapies. K.R. has received grants from the German Federal Ministry of Education and Research (BMBF 01GQ1402, 01DN18022), the German Research Foundation (IRTG 2150, ZUK32/1), Alzheimer Forschung Initiative e.V. (AFI 13812, NL-18002CB) and honoraria for presentations or advisory boards from Biogen and Roche. J.F. is employed by the Friedreich’s Ataxia Research Alliance (FARA) and receives a salary from this institution. L.C. is a research grant recipient from the Friedreich Ataxia Research Alliance (FARA), Ataxia UK, Medical Research Future Fund and is funded by a Medical Research Futures Fund Next Generation Career Development Fellowship. M.B.D. is a research grant recipient from the Friedreich Ataxia Research Alliance (FARA), Medical Research Future Fund and National Health and Medical Research Council. J.B.S. receives grants related to this work from the German Research Foundation (DFG), the German Federal Ministry of Education and Research (BMBF), EuroAtaxia, Voyager Therapeutics, and the Christina Foundation. M.Pan. is a Scientific Advisory Board member of the Friedreich’s Ataxia Research Alliance (FARA), a Board member for the ARSACS Association (Canada), a research grant recipient from FARA, and has consulting/advisory board engagement with Aavanti Bio, Design Therapeutics, Larimar, Minoryx, UCB. M.C. is a co-Founder and Member of the Board of Directors at AavantiBio; is a consultant for Reata Pharmaceutical and AavantiBio; is a member of the Charcot Marie Tooth (CMT) DSMB; is a research grant recipient from the Friedreich’s Ataxia Research Alliance (FARA), Muscular Dystrophy Association (MDA), GOFAR, Duchenne UK foundations and National Institute of Health (NIH)
