# Correction: A natural history study to track brain and spinal cord changes in individuals with Friedreich’s ataxia: TRACK-FA study protocol

**DOI:** 10.1371/journal.pone.0324751

**Published:** 2025-05-22

**Authors:** Nellie Georgiou-Karistianis, Louise A. Corben, Kathrin Reetz, Isaac M. Adanyeguh, Manuela Corti, Dinesh K. Deelchand, Martin B. Delatycki, Imis Dogan, Rebecca Evans, Jennifer Farmer, Marcondes C. França, William Gaetz, Ian H. Harding, Karen S. Harris, Steven Hersch, Richard Joules, James J. Joers, Michelle L. Krishnan, Michelle Lax, Eric F. Lock, David Lynch, Thomas Mareci, Sahan Muthuhetti Gamage, Massimo Pandolfo, Marina Papoutsi, Thiago J. R. Rezende, Timothy P. L. Roberts, Jens T. Rosenberg, Sandro Romanzetti, Jörg B. Schulz, Traci Schilling, Adam J. Schwarz, Sub Subramony, Bert Yao, Stephen Zicha, Christophe Lenglet, Pierre-Gilles Henry

The reference 25 is incorrect. The correct reference is: De Leener B, Levy S, Dupont SM, Fonov VS, Stikov N, Louis Collins D, Callot V, Cohen-Adad J. SCT: Spinal Cord Toolbox, an open-source software for processing spinal cord MRI data. Neuroimage 2017.

In the Author Contribution, Adam J. Schwarz should be credited for the Investigation and Project administration.
