# Supplementary material for: A natural history study to track brain and spinal cord changes in individuals with Friedreich’s ataxia: TRACK-FA study protocol
Source: PLoS One. 2022 Nov 21;17(11):e0269649. doi: 10.1371/journal.pone.0269649 (PMC9678384; doi:10.1371/journal.pone.0269649)
Supplement: Attachment — Submitted filename: Responses to Comments_04MAY2022.docx [file pone.0269649.s001.docx]

**Responses to Issues:**

**Issue 1: Your ethics statement should only appear in the Methods section of your manuscript. If your ethics statement is written in any section besides the Methods, please move it to the Methods section and delete it from any other section. Please ensure that your ethics statement is included in your manuscript, as the ethics statement entered into the online submission form will not be published alongside your manuscript.**

Authors’ revisions in response to Issue 1. Our Ethics Statement is in the Methods section; we have added additional detail to the Ethics Statement, and have moved some details about the consenting process from other sections of the manuscript to the Ethics Statement section, as follows:

**Ethics Statement – Page 12 (of version without tracked changes)**

“*Potential participants will be provided with information about the study via an IRB-approved letter and information sheet.* *Research personnel will explain all study procedures to potential participants. Participants will be enrolled into the study upon obtaining written informed consent and meeting the inclusion and exclusion criteria (see below). Opt-in and opt-out systems are in place for all participants. All participant data will be de-identified upon enrolment into the study using a unique participant identifier. Each site will be required to ensure that participants are consented in such a way that allows the sharing of de-identified data (as outlined in below).*”

**Issue 2: We note that you have indicated that data from this study may be available upon request. PLOS only allows data to be available upon request if there are legal or ethical restrictions on sharing data publicly. Although all articles must include a Data Availability Statement, some submissions, such as Registered Report Protocols and Lab or Study Protocol articles, may not contain data. For manuscripts that do not report data, authors must state in their Data Availability Statement that their article does not report data and the data availability policy is not applicable to their article.**

Authors’ revisions in response to Issue 2. We have clarified that, as a study protocol, our article does not report data; we have moved some text that was previously in the Data Availability Statement to the Data Sharing section of the Methods section, as follows:

**Data Availability – Page 5**

“*This article is a study protocol and does not report data; therefore, the data availability policy is not applicable*.”

**Data sharing – Page 12 (of version without tracked changes)**

“*All parties will have full access to the de-identified raw data throughout the study and interim reporting and final reports will be provided throughout the study in accordance with the milestones and deliverables.*

*All academic partners will provide access to their data to the TRACK-FA Neuroimaging Consortium members and the study funders, as well as the right to create derivative works of the research data*.”

We hope you find our revisions satisfactory and look forward to hearing from you.

Sincerely,

Professor Nellie Georgiou-Karistianis
